# Starving tumor, feeding metastasis: a warning about low-carbohydrate diets in cancer therapy

**DOI:** 10.1038/s41392-025-02469-4

**Published:** 2025-11-21

**Authors:** Byeongsoo Kim, BuHyun Youn

**Affiliations:** 1 Department of Integrated Biological Science, Pusan National University, Busan, Republic of Korea; 2 Department of Biological Sciences, Pusan National University, Busan, Republic of Korea; 3 Nuclear Science Research Institute, Pusan National University, Busan, Republic of Korea

**Keywords:** Metastasis, Tumour immunology

In a recent study published in *Cell*,^1^ Wu et al. reveal that glucose restriction, a promising anti-cancer metabolic therapy, paradoxically promotes lung metastasis by compelling tumor cells to secrete exosomal TRAIL, which establishes an immunosuppressive pre-metastatic niche. This work offers critical new insight into the systemic risks and complex immunological consequences of targeting cancer metabolism and expands our understanding of how primary tumors remotely reshape the microenvironment of distant organs to facilitate metastatic colonization.

**Fig. 1:**
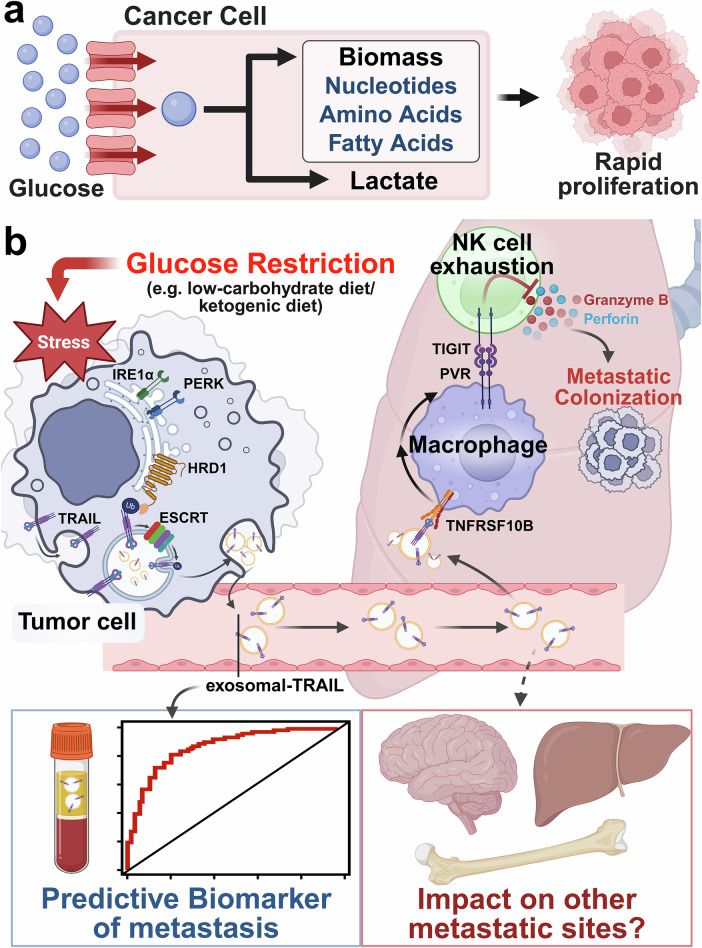
Glucose restriction-induced metastasis: mechanism, biomarker, and future questions **a** The Warburg Effect: Cancer cells exhibit a high rate of glucose uptake, shunting glycolysis intermediates toward the production of lactate and biomass (e.g., nucleotides, amino acids, fatty acids), which serve as biological building blocks to fuel rapid proliferation. **b** A schematic model of the proposed mechanism. (1) Glucose restriction, such as a low-carbohydrate diet, induces ER stress in the primary tumor cell, activating stress sensors like IRE1α and PERK. (2) The ER stress-associated E3 ligase HRD1 is upregulated and catalyzes the K63-linked ubiquitination of TRAIL. (3) The ubiquitinated TRAIL is then recognized and sorted by the ESCRT machinery, leading to its secretion as exosomal TRAIL. (4) These tumor-derived exosomes travel to the lungs, where they engage with their receptor TNFRSF10B on lung macrophages. (5) This interaction activates NF-κB signaling within the macrophages, reprogramming them to express the PVR. (6) The PVR on macrophages engages the TIGIT receptor on lung-resident NK cells. (7) This PVR-TIGIT interaction leads to NK cell exhaustion, suppressing their cytotoxic function and preventing the release of granzyme B and perforin. (8) This depletion of innate immune surveillance establishes an immunosuppressive pre-metastatic niche, facilitating the metastatic colonization of circulating tumor cells. (9) This study identifies plasma exosomal TRAIL as a non-invasive predictive biomarker for metastasis, illustrated by the blood vial and ROC curve. (10) The findings also open future avenues of investigation, such as whether this mechanism impacts other common metastatic sites (e.g., brain, liver, bone). IRE1α, Inositol-requiring enzyme 1 alpha, PERK, Protein kinase R (PKR)-like endoplasmic reticulum kinase, HRD1, HMG-CoA reductase degradation 1, ESCRT, Endosomal sorting complexes required for transport, TRAIL, tumor necrosis factor-related apoptosis-inducing ligand, TNFRSF10B, tumor necrosis factor receptor superfamily member 10B, NF-κB, nuclear factor kappa B, PVR, poliovirus receptor, TIGIT, T-cell immunoreceptor with Ig and ITIM domains, NK cell, natural killer cell. This figure was created with BioRender.com

A cornerstone of cancer biology is the Warburg effect, where tumor cells are metabolically rewired for a profound dependence on glucose to fuel their accelerated proliferation^2^ (Fig. 1a). This has underpinned therapeutic strategies like low-carbohydrate or ketogenic diets, which aim to slow tumor proliferation by “starving” them of one of their primary energy sources. While this approach has shown significant efficacy in reducing primary tumor growth, its influence on tumor progression—the leading cause of cancer mortality—has remained a critical, unresolved question. In a recent Cell publication, Wu and colleagues address this question, beginning with a striking clinical observation that questions the current paradigm. Leveraging the TCGA dataset, they analyzed tumor gene expression from 2514 patients across 22 cancer types. Their analysis revealed a startling paradox: tumors with a low glucose metabolism signature were associated with a significantly higher rate of postoperative recurrence within 2 years across 15 of the cancer types. This was also validated in human hepatocellular carcinoma (HCC), where multimodal analyses, including FDG-PET imaging and LDH activity assays, confirmed the same trend. To determine if this clinical association was causal, the authors turned to a series of in vivo experiments. Across several orthotopic murine tumor models, including hepatoma, melanoma, and breast cancer, a low-carbohydrate diet inhibited primary tumor growth but simultaneously and significantly enhanced lung metastasis. Crucially, this pro-metastatic effect occurred independently of primary tumor size, strongly suggesting that glucose restriction unleashes a distinct, systemic mechanism that actively promotes the colonization of distant organs (Fig. 1b).

The critical question arising from these findings is how a primary tumor under metabolic stress can remotely orchestrate such a pro-metastatic environment in the distant lung. The answer lies in a long-distance communication system mediated by tumor-derived exosomes.^3^ Under glucose deprivation, tumor cells release a significantly increased number of these vesicles. The key to their pro-metastatic message is the protein TRAIL, which the authors show is selectively packaged by a specific molecular machinery. This selective mechanism is driven by endoplasmic reticulum (ER) stress, which activates the ESCRT-dependent pathway to sort K63-linked ubiquitinated TRAIL into the exosomes for export.^4^ Once secreted into the bloodstream, these TRAIL-carrying exosomes journey to the lungs and deliver their signal primarily to lung macrophages. The arrival of this signal triggers NF-κB signaling within the macrophages, fundamentally reprogramming them from potential defenders into active facilitators of metastasis,^5^ marked by high expression of the poliovirus receptor (PVR). The newly PVR-expressing macrophages engage with the TIGIT receptor on the lung’s natural killer (NK) cells—which are critical for innate anti-tumor immunity—ultimately neutralizing the NK cell population through exhaustion and depletion. This final step dismantles the lung’s innate immune surveillance, creating an immunosuppressive, pre-metastatic niche where circulating tumor cells can now easily colonize.

The discovery of this double-edged sword effect of glucose restriction on cancer treatment immediately raises two clinical questions: how can one detect this pro-metastatic risk in patients, and how can the danger be neutralized to safely harness the diet’s anti-tumor benefits? Wu and colleagues provide clear, mechanism-based answers to both. To neutralize the danger, they specifically targeted the PVR-TIGIT signaling axis. Their results showed that blocking TIGIT with antibodies not only completely abrogated glucose-restricted diet-induced lung metastasis but also synergistically enhanced primary tumor regression. This presents a powerful therapeutic strategy to uncouple the anti-tumor effects of glucose restriction from its pro-metastatic consequences. Furthermore, the study provides a vital tool for detecting this risk in advance. The authors identified plasma exosomal TRAIL as a non-invasive biomarker. In their HCC validation cohort, it predicted early postoperative lung metastasis with remarkable precision, achieving an area under the receiver operating characteristic curve of 0.824 for 1-year prediction, significantly outperforming traditional markers. This suggests a powerful clinical paradigm: using exosomal TRAIL to identify high-risk HCC patients who are ideal candidates for a combination of metabolic therapy and TIGIT immunotherapy. Based on this finding, it would be valuable to determine if exosomal TRAIL can also serve as a biomarker across other tumor types prone to lung metastasis.

The work by Wu and colleagues is a landmark study that serves as a crucial warning for the emerging field of cancer metabolic therapy. It fundamentally challenges the simple paradigm of “starving the tumor,” compelling the field to consider the intricate and often counterintuitive interplay between cancer metabolism and systemic immunity. While the study dissects the pro-metastatic mechanism in the lung, it opens up several avenues for future investigation. Does glucose restriction exert similar pro-tumorigenic effects in other common metastatic sites? Could other dietary interventions also systemically influence the pre-metastatic niche and hence cancer progression? Furthermore, does this “feeding” effect extend beyond metastasis to promote other malignant phenotypes, such as chemo-/radio-therapy resistance or cancer cell plasticity? These questions underscore what is perhaps the broadest implication of this work: the compelling challenge it poses to a one-size-fits-all approach to diet in oncology. The study clearly demonstrates that a systemic metabolic intervention like a low-carbohydrate diet is not simply “good” or “bad,” but can have profoundly different, even opposing, effects on primary tumor growth versus metastatic progression. Therefore, the future of nutritional oncology may require a paradigm shift: moving away from the general question of “which diet is best for cancer?” and toward a more personalized approach that asks, “for which patient, at what stage of disease, and in combination with which therapy?” In this respect, Wu et al. suggest that a patient’s tumor metabolic signature, or the level of a biomarker, could one day be used to guide such personalized dietary recommendations. Indeed, this research highlights a pressing need to shift the field’s focus toward systemic tumor–host crosstalk and the broader macroenvironment. Understanding this complex metabolic interplay is an emerging frontier with strong potential to advance cancer diagnosis and therapy. The work by Wu et al. provides a valuable example of this new direction, reminding us that the outcome of a metabolic therapy is not absolute, but is dictated by its complex and often unexpected interplay with the broader host physiology.
